# The autonomic balance of heart rhythm complexity after renal artery denervation: insight from entropy of entropy and average entropy analysis

**DOI:** 10.1186/s12938-022-00999-4

**Published:** 2022-05-24

**Authors:** Po-Lin Lin, Ping-Yen Lin, Han-Ping Huang, Hamideh Vaezi, Lawrence Yu-Min Liu, Ying-Hsiang Lee, Chun-Che Huang, Ten-Fang Yang, Long Hsu, Chang Francis Hsu

**Affiliations:** 1 Division of Cardiology, Hsinchu MacKay Memorial Hospital, Hsinchu, Taiwan; 2 Department of Biological Science and Technology, National Yang Ming Chiao Tung University, Hsinchu, Taiwan; 3 Department of Electrophyics, National Yang Ming Chiao Tung University, Hsinchu, Taiwan; 4 Labfront (Kiipo Co.), Boston, USA; 5 Center for Academia-Industry Collaboration, National Yang Ming Chiao Tung University, Hsinchu, Taiwan; 6 Department of Medicine, MacKay Medical College, New Taipei, Taiwan; 7 Department of Artificial Intelligence and Medical Application, MacKay Junior College of Medicine, Nursing, and Management, Taipei, Taiwan; 8 Cardiovascular Center, MacKay Memorial Hospital, Taipei, Taiwan; 9 Department of Healthcare Administration, College of Medicine, I-Shou University, Kaohsiung, Taiwan; 10 Graduate Institute of Medical Informatics, Taipei Medical University and Hospital, Taipei, Taiwan

**Keywords:** Renal denervation (RDN), Autonomic balance, Heart rate variability (HRV), Disorder, Complexity

## Abstract

**Background:**

The current method to evaluate the autonomic balance after renal denervation (RDN) relies on heart rate variability (HRV). However, parameters of HRV were not always predictive of response to RDN. Therefore, the complexity and disorder of heart rhythm, measured by entropy of entropy (EoE) and average entropy (AE), have been used to analyze autonomic dysfunction. This study evaluated the dynamic changes in autonomic status after RDN via EoE and AE analysis.

**Methods:**

Five patients were prospectively enrolled in the Global SYMPLICITY Registry from 2020 to 2021. 24-h Holter and ambulatory blood pressure monitoring (ABPM) was performed at baseline and 3 months after RDN procedures. The autonomic status was analyzed using the entropy-based AE and EoE analysis and the conventional HRV-based low frequency (LF), high frequency (HF), and LF/HF.

**Results:**

After RDN, the ABPM of all patients showed a significant reduction in blood pressure (BP) and heart rate. Only AE and HF values of all patients had consistent changes after RDN (*p* < 0.05). The spearman rank-order correlation coefficient of AE vs. HF was 0.86, but AE had a lower coefficient of variation than HF.

**Conclusions:**

Monitoring the AE and EoE analysis could be an alternative to interpreting autonomic status. In addition, a relative change of autonomic tone, especially an increasing parasympathetic activity, could restore autonomic balance after RDN.

**Supplementary Information:**

The online version contains supplementary material available at 10.1186/s12938-022-00999-4.

## Background

Autonomic imbalance, characterized by a hyperactive sympathetic system and a hypoactive parasympathetic system, is associated with various pathological conditions [1]. Conventionally, the relationships between autonomic imbalances and cardiovascular diseases are widely implemented by analyzing the heart rate variability (HRV) in frequency domain. The low-frequency (LF) spectral power of a heart rate signal within the spectra between 0.04 Hz and 0.15 Hz reflects both the sympathetic and parasympathetic activities. On the other hand, the high-frequency (HF) spectral power between 0.15 Hz and 0.4 Hz is considered a reflection of solely parasympathetic activity. The corresponding LF/HF ratio is often used to indicate the autonomic balance [1–4]. Consequently, the autonomic imbalance measured by HRV has been shown to be associated with a wide range of pathological conditions, including congestive heart failure (CHF), diabetes, osteoporosis, arthritis, Alzheimer’s disease, periodontal disease, and hypertension (HTN) [1–5].

In practice, the HRV analysis has been attempted to assess the effectiveness of percutaneous renal denervation (RDN), an effective and safe treatment to reduce blood pressure (BP) by disrupting renal sympathetic nerves for the autonomic balance [6, 7]. Applying the short-term HRV after RDN or renal nerve stimulation seems to provide a way to assess the state of the autonomic nervous system [8, 9]. However, the relative changes of HRV after RDN were not always consistent with BP reduction and were not predictive of response to RDN [10–12]. The mechanisms of non-responder after RDN was uncertain, and the possible cause was the preexisting activity of the sympathetic nervous system before RDN [13]. Furthermore, the indices of conventional HRV could be influenced by different backgrounds, exercise, or unplanned medication changes and had a broad baseline value [14–16]. Another limitation of conventional HRV indices is that both LF and LF/HF reflect the mix of sympathetic and parasympathetic activities. Thus, it is difficult to discern the underlying meaning [14, 15].

Later, some conditional-entropy-based methods provided another way to detect autonomic dysfunction by analyzing the irregularity of heart rhythm. For example, approximate entropy (ApEn) and sample entropy (SampEn) measure the irregularity of a heart rate time series by evaluating the appearance of repetitive patterns in the series [17, 18]. However, the ApEn might underestimate the actual irregularity due to the self-counting of unique patterns. In addition, SampEn would blur the local statistical features while computing over a global estimate of conditional probability. To overcome the disadvantages of ApEn and SampEn, corrected conditional entropy (CCE) and local SampEn (LSampEn) were proposed [19, 20]. The CCE of heart rate series has been shown to decrease as a function of the tilt table inclination and thus can be helpful to monitor sympathovagal balance [19, 21]. LSampEn of heart period variability has been shown to decrease during bicycle exercise, thus being a likely hallmark of sympathetic activation [20].

Although the conventional HRV indices and the conditional-entropy-based irregularities quantify autonomic dysfunction from different viewpoints, there are various drawbacks. For example, the frequency-domain HRV measures the spectral power of a time series, which reflects the amplitude of the series without considering the sequential irregularity of the series. On the other hand, the conditional-entropy-based irregularities neglect the amplitude of the series, since the standard deviation of the series is always normalized to 1 in their algorithms.

Recently, the disorder of heart rhythm measured using average entropy (AE) seems superior to the irregularity in terms of the accuracy in differentiating the healthy from the pathological. Average entropy (AE) measures the disorder of a time series by reflecting both the amplitude and randomness of the series [22]. AE is the average of local instabilities of a series measured using multi-scale Shannon entropy instead of evaluating the appearance of repetitive patterns of the series. AE has been shown reliable in quantifying the disorders of simulated series with different amplitudes and numbers of shuffled data points [22] and those of colored noises [23]. In the heart rate signal analysis, AE has been used to differentiate the healthy, the congestive heart failure (CHF), and the atrial fibrillation (AF) subjects with an accuracy of 94%, higher than those obtained using Shannon entropy and SampEn.

In addition, the complexity of heart rhythm measured using entropy of entropy (EoE) reflects the degree of healthiness [24]. The EoE vs. AE plot of a group of heart rate series exhibits a distinct inverted U relation. The corresponding data points from the CHF, the healthy, and the AF subjects can be well-separated in the lower left, middle top, and lower right regions. Specifically, the healthy subjects were found highly converged in the area of 1.0 ≤ AE ≤ 1.8 and EoE ≥ 3.8 [22].

Since it has been well-recognized that CHF and AF are associated with autonomic dysfunction, the sympathetic activities and autonomic balance changes are likely to be reflected in the AE and EoE analysis. This might provide an alternative way to detect autonomic dysfunction. Therefore, this study aimed to investigate the role of RDN in restoring autonomic balance via AE and EoE analysis.

## Results

### Change of blood pressure and HRV after RDN

A total of five patients, four males and one female, with resistant HTN were enrolled in this pilot study. Their mean age was 48.6 ± 11.3 years. Table 1 shows the statistical features of their ambulatory BP monitoring (ABPM) data at baseline (before RDN) and 3 months after RDN. At baseline, the means of the 24-h systolic BP (SBP) and diastolic BP (DBP) of the 5 subjects were 144.0 mmHg and 90.2 mmHg. Three months after RDN, all the SBPs and the DBPs were both reduced, including daytime and nighttime periods.

**Table 1:** Changes in ABPM in patients treated RDN after 3 months

	Before (*t*_1_)	After (*t*_2_)	Difference (*t*_2−_t_1_)
24-h SBP (mmHg)	144.0 (121 ~ 173)	124.8 (109 ~ 155)	−19.2 (−40 to −12)
daytime SBP (mmHg)	147.8 (135 ~ 170)	125.6 (114 ~ 128)	−22.2 (−45 to −7)
night SBP (mmHg)	136.0 (104 ~ 147)	121.0 (98 ~ 153)	−15 (−38 to −6)
24-h DBP (mmHg)	90.2 (76 ~ 105)	77.6 (65 ~ 85)	−12.6 (−29 to −3)
daytime DBP (mmHg)	92.0 (87 ~ 107)	78.6 (72 ~ 86)	−13.4 (−28 to 2)
night DBP (mmHg)	84.6 (59–95)	71.4 (50–84)	−10.8 (−25 to −6)

**Fig. 1:**
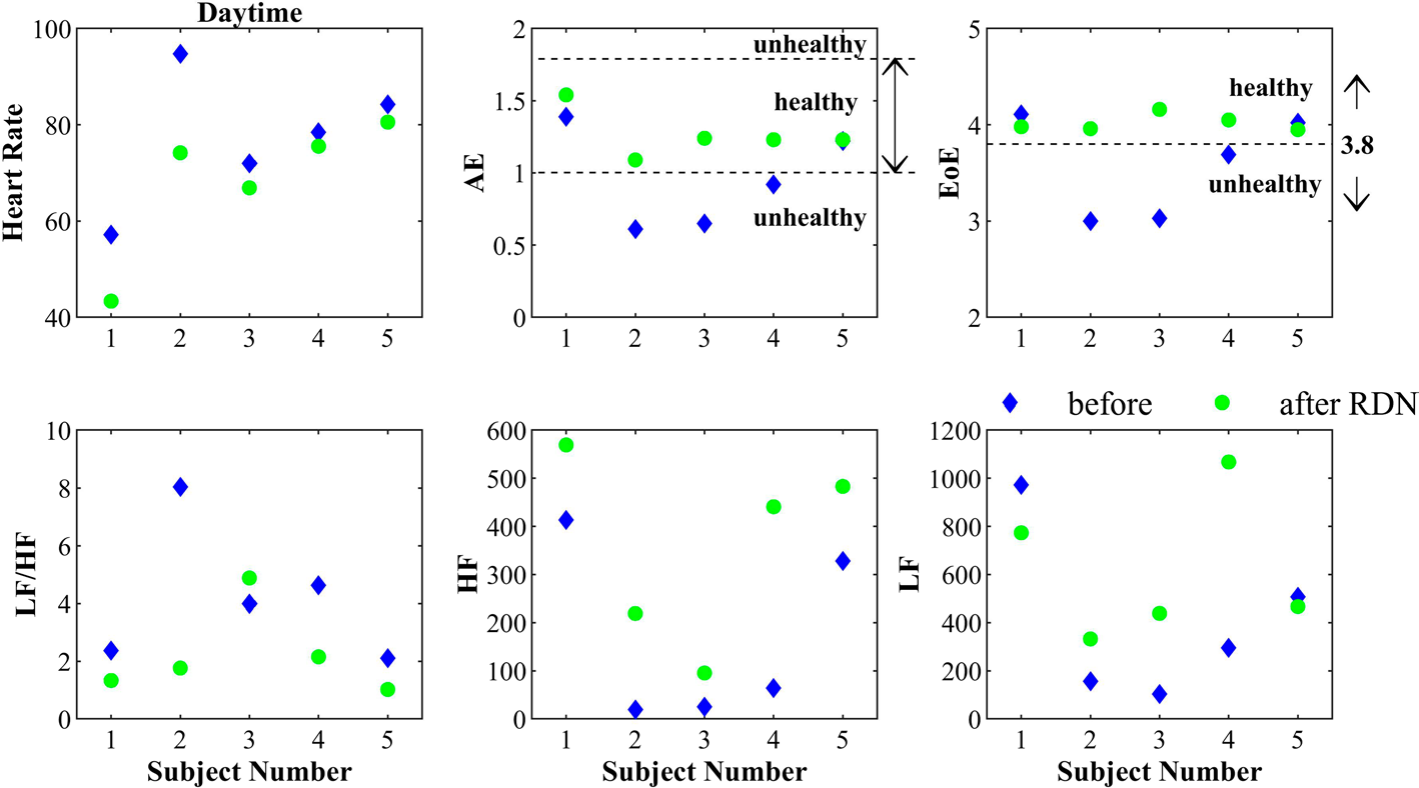
Daytime average values of the six HRV indices: before (blue diamond) and after (green circle) RDN. The health threshold was defined according to previous analysis [15]

Figure 1 illustrates the average daytime heart rate, AE, EoE, LF/HF, HF, and LF of all 1-h RR interval series before and after RDN of all subjects. The heart rates of all subjects decreased after RDN. Only the AE and HF values of all subjects consistently increased after RDN. The LF/HF ratio as an index of autonomic balance did not have a consistent change after RDN. The post-RDN values of AE and EoE were distributed within a relatively small range compared with other HRV indices. According to previous analysis [22], the post-RDN values of AE and EoE were all within the threshold of being healthy status (1.0 ≤ AE ≤ 1.8 and EoE ≥ 3.8).

The post-RDN values of AE and EoE had a lower coefficient of variation (CV) than other HRV indices (Table 2). Lower CV values of AE and EoE resulted in narrow distribution after RDN, as shown in Fig. 1.

**Table 2:** Coefficient of variation (CV) of six HRV indices from the 5 subjects (*N* = 5): before and after RDN

	Before RDN (*N* = 5)		After RDN (*N* = 5)		*p*
	Mean ± SD	CV	Mean ± SD	CV	
Heart rate (min^−1^)	77.31 ± 14.026	0.18	68.11 ± 14.67	0.22	< 0.05
LF (ms^2^)	407.18 ± 352.67	0.87	615.87 ± 301.17	0.49	0.35
HF(ms^2^)	169.98 ± 186.48	1.10	361.52 ± 197.03	0.55	< 0.05
LF/HF	4.23 ± 2.38	0.56	2.23 ± 1.54	0.67	0.08
AE	0.96 ± 0.34	0.36	1.26 ± 0.17	0.13	< 0.05
EoE	3.57 ± 0.53	0.15	4.02 ± 0.09	0.02	0.23

### Correlation between HRV indices

**Fig. 2:**
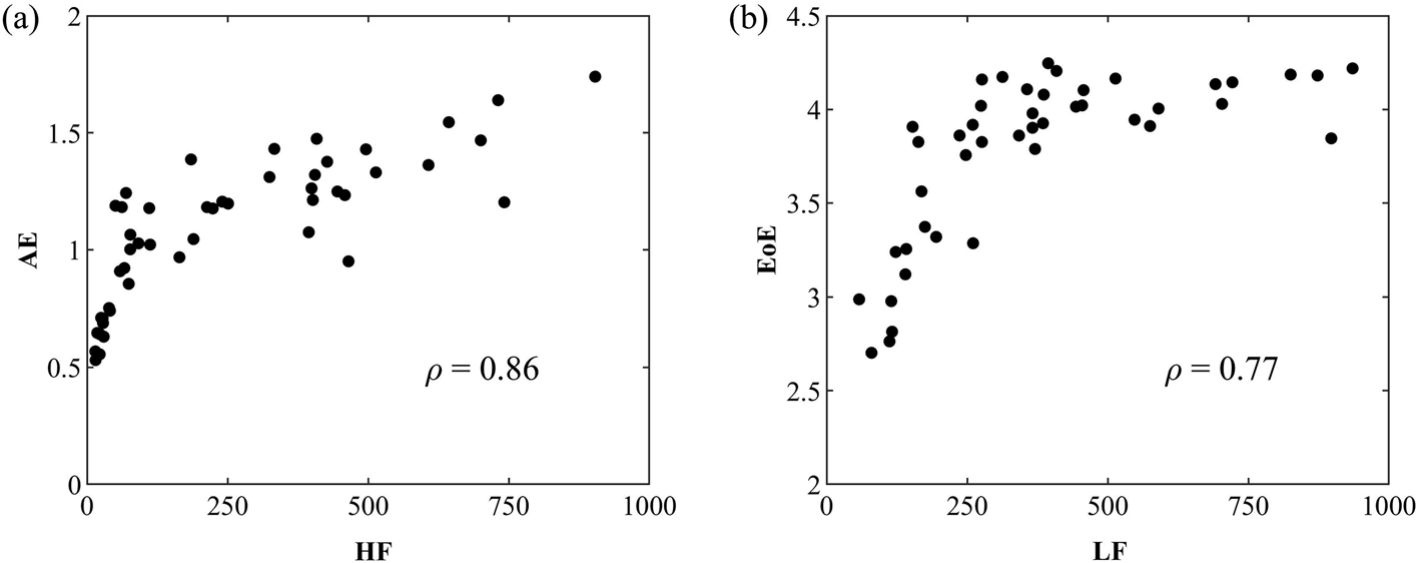
Corresponding Spearman rank-order correlation (*ρ*) coefficients of **a**. AE vs. HF and **b** EoE vs. LF of all 48 RR daytime interval series, including before and after RDN

Table 3 illustrates the Spearman rank-order correlation (*ρ*) coefficients between any two HRV indices. The AE and EoE were strongly correlated with the HF and LF, respectively. Figure 2 shows (a) the AE vs. HF and (b) the EoE vs. LF of 48 1-h RR daytime interval series, including before and after RDN. The spearman rank-order correlation (*ρ*) coefficient of (a) the AE vs. HF and (b) the EoE vs. LF are 0.86 and 0.77, respectively.

**Table 3:** Spearman rank-order correlation (*ρ*) coefficients between any two HRV indices

	HF	LF/HF	AE	EoE
LF	0.78	−0.30	0.79	0.77
HF		−0.76	0.86	0.60
LF/HF			−0.56	−0.28
AE				0.73

### EoE vs. AE of the heart rate signals before and after RDN

**Fig. 3:**
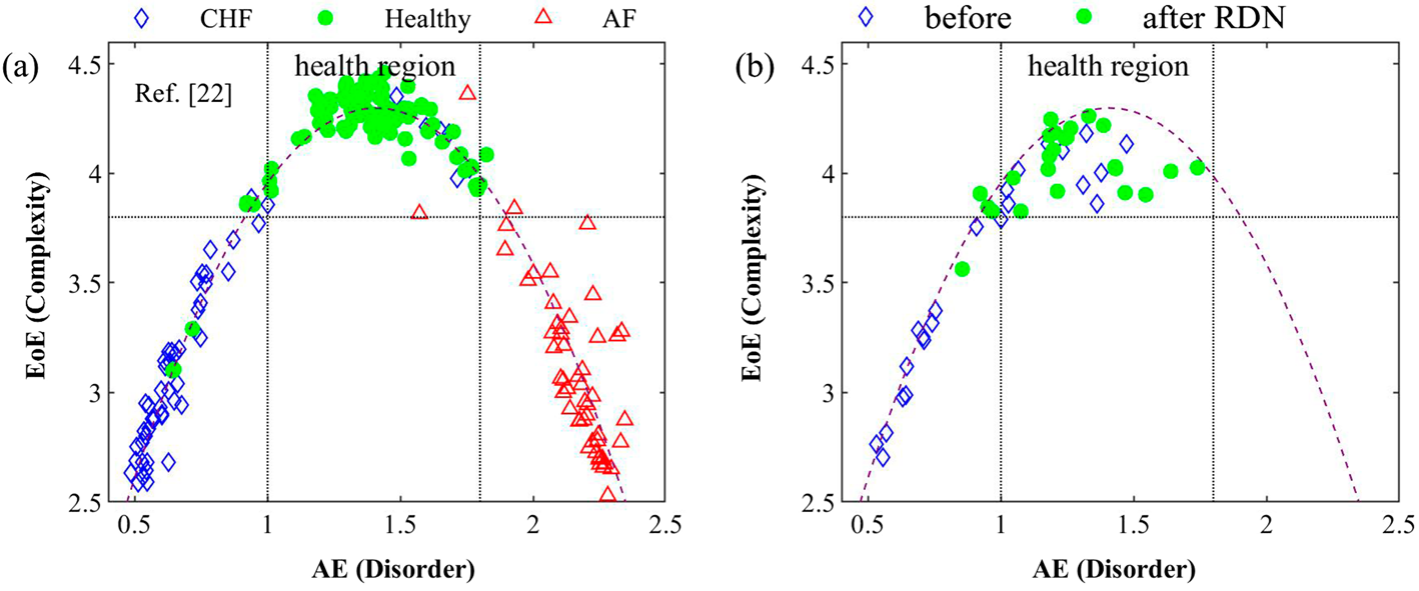
**a** Inverted U shape of the EoE vs. AE plot from the previous study [22]. The diamond, circle, and triangle symbols are from 15 CHF, 18 healthy, and 53 AF subjects, respectively. The dashed curve is a quadratic fitting in each plot. **b** EoE vs. AE of all 48 1-h RR interval series before (blue diamonds) and after (green circles) RDN from the five subjects in this study

Figure 3a illustrates the inverted U shape of the EoE vs. AE plot from the previous study [22], in which the dashed curve is a quadratic fitting to the data as a reference in Fig. 3b. The data is from 75 CHF, 90 healthy, and 53 AF sets of heart rate series with 10,000 data points. The data points from the healthy set are highly concentrated in the region of 1.0 ≤ AE ≤ 1.8 and EoE ≥ 3.8. Figure 3b illustrates the EoE vs. AE plot of all 48 1-h RR interval series before (blue diamonds) and after (green circles) RDN from the 5 subjects in this study. It can be seen that the data shifted from the lower-left region before RDN to the middle-top region after RDN, corresponding to the CHF and health regions, as shown in Fig. 3a.

## Discussion

As shown in Fig. 3b, the EoE and AE data shift after RDN toward the health zone provides an interpretation of EoE and AE on the autonomic balance of heart rhythm complexity after renal artery denervation. First, a review of the nature of the disorder and complexity of the RR interval series, measured using AE and EoE [22, 24], is beneficial to understanding the inverted U relation in the EoE vs. AE plot of heart rhythm. The RR series from CHF patients exhibit relatively stable behavior, while those from AF patients show highly erratic fluctuations with statistical properties resembling uncorrelated white noise. Thus, the AE values of AF, healthy, and CHF groups are high, medium, and low, respectively. Furthermore, AE has been shown to have higher accuracy in differentiating the three groups than Shannon entropy and sample entropy [22].

On the other hand, the complexity measured using EoE has been illustrated as relatively high for a complex system intermediate between extreme order and disorder [22–27]. In other words, complexity is considered different from disorder or randomness. The complexity of RR interval series is regarded as a reflection of the degree of the healthiness of heart rhythm [28]. Thus, the EoE value is significantly higher in a healthy group than in CHF and AF groups. Furthermore, EoE has been shown to have higher accuracy in differentiating the healthy from the pathologic groups than the multi-scale entropy [24].

Thus, the EoE vs. AE plot of the RR series displays an inverted U relation. The corresponding data from CHF, healthy, and AF subjects lie in the lower-left, middle-top, and lower-right regions, respectively, as mentioned in the introduction section. The feature is consistent with the hypothesis in the previous studies [28–32]. Thus, the combination of EoE and AE, such as the inverted U relation, could be an alternative method to evaluate autonomic balance instead of the traditional LF/HF ratio.

Second, recall that CHF and hypertension (HTN) are related to an autonomic imbalance with increased sympathetic output and decreased parasympathetic tone [1, 33]. In this study, the (EoE, AE) data of HTN patients were found to shift from the lower-left region to the middle-top region after RDN, corresponding to the CHF and health regions in the previous study [22]. Therefore, this result suggests that the healthy and pathologic status zones created by the EoE vs. AE plot could be applied to diagnose the clinical diseases due to autonomic dysfunction.

From another point of view, as shown in Fig. 2a, the large Spearman rank-order correlation coefficient of AE vs. HF, 0.86, also suggests that AE might be an effective indicator of evaluating parasympathetic nerve activity as HF. After RDN, the mean AE was increasing from 0.96 ± 0.34 to 1.26 ± 0.17 (*p* < 0.05). The change of AE is consistent with the previous studies in which the entropy values increase as the antinomic balance is increased [19–21]. The changes of AE might reflect an increasing parasympathetic tone, while RDN suppressed the over-activated sympathetic tone. In conventional HRV analysis, LF was an index of sympathetic cardiac control.

Similarly, as shown in Fig. 2a, the large Spearman rank-order correlation coefficient of EoE vs. LF, 0.77, also implies that EoE might be an effective index of autonomic balance as LF. After RDN, the mean EoE was increased from 3.57 ± 0.53 to 4.02 ± 0.09. The changes in EoE might reflect a decreasing sympathetic tone after RDN. The post-RDN (EoE, AE) data points were shifted toward the relative health region, the middle-top of the inverted U plot. Although the LF/HF ratio was an index of autonomic balance, the post-RDN LF/HF ratio did not show a consistent decreasing change in individual patients. The mean LF/HF ratio decreased from 4.23 ± 2.38 to 2.23 ± 1.54, but its coefficient of variation (CV) was higher than AE and EoE. In our study, the post–RDN data of AE and EoE were all within the relative health region: (1) 1.0 ≤ AE ≤ 1.8 and (2) EoE ≥ 3.8. Consequently, the top of the inverted U shape established by AE and EoE could better evaluate autonomic balance.

## Conclusions

The autonomic imbalance of excessive sympathetic activity and reduced parasympathetic activity represents a significant risk for cardiovascular mortality. Compared with the conventional HRV, the complexity and disorder of heart rhythm, measured using EoE and AE, provide an alternative method of interpreting sympathetic and parasympathetic status. After disrupting renal sympathetic nerves by RDN, AE and EoE converged into an area (1.0 ≤ AE ≤ 1.8 and EoE ≥ 3.8), the health zone [22]. This result implies that the healthy and pathologic status zones created by the EoE vs. AE plot could be applied to diagnose the clinical diseases due to autonomic dysfunction.

Besides, the relative change of autonomic tone, especially an increasing parasympathetic activity, could restore autonomic balance after RDN**.** Since there is a high correlation between AE and HF, AE might also be an effective indicator of parasympathetic activity.

## Materials and methods

### Patients

This analysis is based on five patients who participated in the Global SYMPLICITY Registry (GSR), who underwent RDN from March 11, 2020, to February 18, 2021 [34]. The GSR is an ongoing, multicenter, international single-arm trial with a planned enrollment of 3000 patients with uncontrolled HTN and/or conditions associated with sympathetic nervous system activation. Uncontrolled HTN was defined as BP above-recommended levels (regardless of therapy) according to published local guidelines. Sympathetic nervous system activation was defined as conditions associated with increased sympathetic nervous system activity, including diabetes, CHF, chronic kidney disease, obstructive sleep apnea, or arrhythmias.

Eligible patients were older than 18 years and fulfilled one of the criteria: (1) office systolic BP ≥ 150 mmHg and diastolic BP ≥ 90 mmHg (2) ambulatory systolic BP ≥ 140 mmHg and diastolic BP ≥ 80 mmHg type 2 diabetics, despite treatment with ≥ 3 antihypertensive drugs with glomerular filtration rate ≥ 45 mL/min/1.73 m^2^ and suitable renal artery anatomy for RDN. The Institutional Review Board of Mackay Memorial Hospital approved the study protocol (number 19CT035Ae).

### Ambulatory blood pressure monitoring (ABPM) and sample preparation

The ABPM readings of the 5 subjects were obtained at baseline and 3 months after RDN, separately. The ABPM was performed using the WatchBP Home device (Microlife Inc., Widnau, Switzerland) with readings taken every 30 min at daytime and every 60 min at nighttime (daytime: 08:00 to 18:00, nighttime: 22:00 to 04:00 [35, 36]). Further examinations included complete history, assessment of office BP and 24-h ABPM, review of medication, and blood chemistry. Bilateral RDN was analyzed using the Symplicity Spyral catheter (Medtronic, Mountain View, California). All procedures were performed by interventionists smoothly without any complications.

### Electrocardiography (ECG) monitoring and sample preparation

The 24-h Holter monitoring for the ECG data of the 5 subjects was performed before and immediately after RDN procedures. The ECG data collected from the 5 subjects were prepared for HRV analysis as follows. First, each of the 5 sets of ECG data was converted into a heart rate (RR interval) time series. Second, the daytime data segments of the RR interval series during the daytime periods were extracted (08:00 to 18:00). Third, each of the daytime data segments was truncated into several sets of 1-h-long time series. This was to examine the variations of the 5 HRV indices with time for each of the 5 subjects.

Regarding the daytime samples after RDN for analysis, there was an average of approximately 5 1-h-long RR interval time series from each of the 5 subjects from 13:00 to 18:00, since all the 5 RDN procedures were ended around 12:00 in the afternoon. In total, there were 24 sets of 1-h-long RR interval time series after RDN. Accordingly, 24 sets of daytime 1-h-long RR time series before RDN were thus prepared for analysis.

### HRV analyses

The conventional HRV indices LF, HF, and LF/HF, and the entropy-based EoE and AE of each 1-h-long RR interval time series were evaluated. Here, the LF is the spectral power of a RR interval time series in the low-frequency band between 0.04 to 0.15 Hz. The HF is the spectral power of the RR interval time series in the high-frequency band from 0.15 to 0.4 Hz.

### Entropy of entropy (EoE) and average entropy (AE) methods

EoE characterizes the complexity of a biological system from the viewpoint of the "variation of information" hidden in a physiologic time-series signal on multiple time scales [24]. AE reflects the disorder of a time series in terms of both amplitude and randomness of the series on multiple time scales [22].

The algorithms of both EoE and AE methods consist of three steps in analyzing a time series {*x*_*i*_} = {*x*_1_, …, *x*_*N*_} of length *N*. The first and the second steps of the two methods are the same for the construction of a Shannon entropy sequence to represent the time series {*x*_*i*_}. First, the time series is divided into many consecutive non-overlapping windows of equal length*τ*. Each window is in the form of *w*_*j*_^(*τ*)^ = {*x*_(*j−*1)τ+1_, …, *x*_(*j*−1)τ+τ_}, where *j* is the window index ranging from 1 to *N*/*τ* and *τ* corresponds to the scale factor of EoE and AE.

Second, the Shannon entropy value of each window *w*_*j*_^(*τ*)^ is derived as follows. Suppose that *x*_max_ and *x*_min_ are the maximum and minimum of all data collected in this study, respectively. The range from *x*_max_ to *x*_min_ is divided into *s*_1_ slices of equal width Δ*s*_1_ = (*x*_max_ − *x*_min_)/*s*_1_. The probability *p*_*jk*_ for a certain data point *x*_*i*_ over window *w*_*j*_^(*τ*)^ to occur in slice *k* is thus obtained in the form of

1 $$p_{jk} = \frac{{{\text{total number of }}x_{i} {\text{over }}w_{j}^{\left( \tau \right)} {\text{in slice }}k}}{\tau }$$

where *k* is the slice index ranging from 1 to *s*_1_. Subsequently, the Shannon entropy value y_j_^(*τ*)^ of each window *w*_*j*_^(*τ*)^ is given by

2 $$y_{j}^{\left( \tau \right)} = - \mathop \sum \limits_{k = 1}^{{s_{1} }} p_{jk} \left( { {\text{In}} p_{jk} } \right)$$

In this respect, the Shannon entropy value *y*_*j*_^(*τ*)^ is considered the representative of window *w*_*j*_^(*τ*)^. Repeating the same process for every window results in a representative Shannon entropy sequence {*y*_*j*_^{*τ* }^} of length *N*/*τ* for the original time series {*x*_*i*_}.

Third, the AE value of {*x*_*i*_} is defined as the average of the Shannon entropy sequence {*y*_*j*_^*(τ)*^} in the form of

3 $${\text{AE}}\left( \tau \right) = \frac{{\mathop \sum \nolimits_{j = 1}^{N/\tau } y_{j}^{\left( \tau \right)} }}{N/\tau }$$

On the other hand, the EoE value of {*x*_*i*_} is derived as follows. It can be imagined that all elements of {*y*_*j*_^{*τ*}^} distribute over some finite levels and the maximum number of all possible levels *s*_2_ (*τ*) depends upon the time scale *τ*. For example, *s*_2_ (1) = 1, *s*_2_ (2) = 2, *s*_2_ (3) = 3, *s*_2_ (4) = 5, *s*_2_ (5) = 7, and *s*_2_ (6) = 11. Then, the probability *p*_*l*_ for a certain representative *y*_*j*_^{*τ*}^ over the sequence {*y*_*j*_^(*τ*)^} to occur in level *l* is obtained in the form of

4 $$p_{l} = \frac{{total number of y_{j}^{\left( \tau \right)} over \left\{ {y_{j}^{\left( \tau \right)} } \right\} in level l}}{N/\tau }$$

where *l* is the level index ranging from 1 to *s*_2_. Thus, the EoE value of the original time series {*x*_*i*_} is defined as the Shannon entropy value of the Shannon entropy sequence {*y*_*j*_^(*τ*)^} and is given by.

5 $${\text{EoE}}\left( \tau \right) = - \mathop \sum \limits_{l = 1}^{{s_{2} }} p_{l} \left( {{\text{ln}}p_{l} } \right).$$

In this study, *x*_max_ = 1.6, *x*_min_ = 0.3, *τ* = 14, and *s*_1_ = 55 are used for the RR interval series analysis, as suggested in the previous study [22].

In this paper, the entropy-based AE and EoE analysis is proposed to test the hypothesis of whether RDN is effective in restoring the balance of the autonomic system. The criteria for AE and/or EoE to be the valid indicators of sympatho-vagal balance are as follows: (1) the values of the indicators after RDN should separately range within a health region for all test subjects, and (2) the values of the indicators after RDN should demonstrate the lowest variabilities among the subjects [37]. In this study, the two criteria were tested for AE, EoE, and the conventional HRV indices LF, HF, and LF/HF, separately, for comparison by analyzing the BP and the ECG data of 5 resistant HTN patients enrolled. Among the five indices, the ones that best satisfy the criteria would be the ideal indicators.

### Examples of the AE and the EoE analyses of heart rate signals and the inverted U relation

**Fig. 4:**
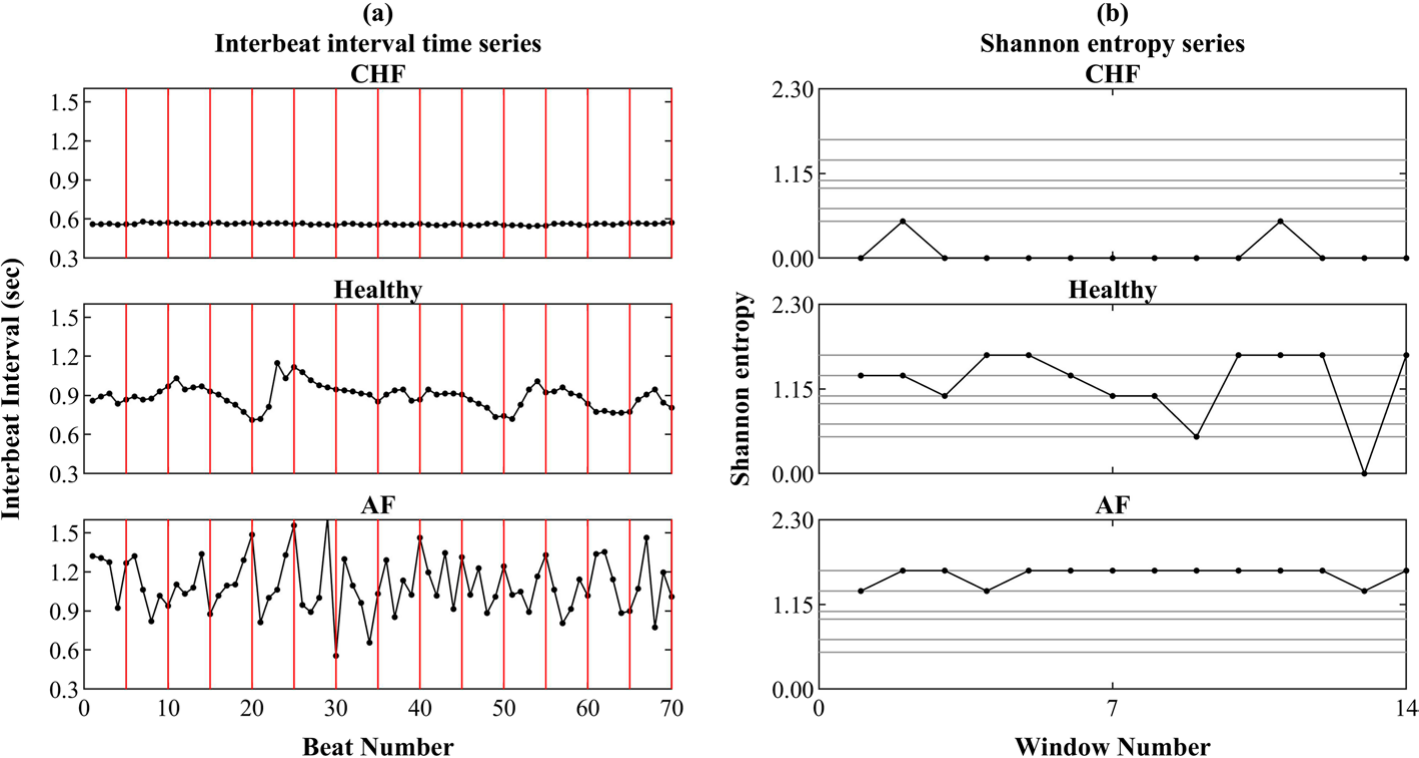
**a** Three RR interval series {x_i_} with the same length of 70 data points. **b** Shannon entropy sequences {*y*_*j*_^(5)^} of the three heart rate series for EoE and AE analyses at *τ* = 5 as an example of short data analysis

Figure 4 illustrates the three steps of the AE and EoE methods for three representative CHF, healthy, and AF time series of consecutive heartbeat intervals. Figure 4a shows the three RR interval series {*x*_*i*_} with the same length *N* of 70 data points. In this example case, all series were analyzed at *τ* = 5. It can be seen that the 70 data points of each noise were equally divided into 16 (= *N*/*τ*) windows, with each of 5 data points in a red frame. Then, the Shannon entropy value of every window in red was calculated individually. Figure 4b shows the representative Shannon entropy sequences {*y*_*j*_^(5)^} of the three RR series. Each sequence {*y*_*j*_^(5)^} consists of 16 elements, separately. According to Eqs. (5) and (3), the (EoE, AE) values of the three noises were obtained to be (0.41, 0.07), (1.40, 1.24), and (0.41, 1.57), individually. It can be seen that EoE is maximum for healthy subjects with intermediate AE, and thus the EoE vs. AE plot exhibits an inverted U shape.

### Statistical analysis

The BP data were presented as mean (min.–max.). The HRV data were presented as mean ± standard deviation (SD). The variability of each HRV index among a specific set of samples was expressed as the coefficient of variation of the values of the HRV index. The correlation between any two different HRV indices among a specific set of samples was evaluated using the Spearman rank-order correlation method. The degree of difference between the before RDN and the after RDN samples for each HRV index was performed using the paired Wilcoxon signed-rank test (which is suitable for small sample comparison based on nonparametric statistics). The statistical significance was set at *p* < 0.05. All data analyses were performed using IBM SPSS 22.0 for windows (IBM Corp., Chicago, IL, USA).

## Supplementary Information

**Additional file 1.** RightsLink Printable License. In Figs. 1 and 3, we reused the dashed auxiliary lines and EoE vs. AE plot from our previous study published in Physica A to further explore whether the new result is consistent with the previous one. We explained and cited the work in the text and attached the RightsLink Printable License.

## Data Availability

Not applicable.

## Abbreviations

RDN: Renal denervation
HRV: Heart rate variability
EoE: Entropy of entropy
AE: Average entropy
ABPM: Ambulatory blood pressure monitoring
BP: Blood pressure
HTN: Hypertension
LF: Low frequency
HF: High frequency
SD: Standard deviation
CV: Coefficient of variation
